# Effect of exercise intensity and blood glucose level on glucose requirements to maintain stable glycaemia during exercise in individuals with type 1 diabetes

**DOI:** 10.1186/1687-9856-2015-S1-O39

**Published:** 2015-04-28

**Authors:** Shetty Vinutha, Fournier Paul, Davey Raymond, Retterath Adam, Roby Heather, Paramalingam Nirubasini, Cooper Matthew, Davis Elizabeth, Jones Timothy

**Affiliations:** 1 Department of Endocrinology and Diabetes, Princess Margaret Hospital, Subiaco, WA, Australia; 2 School of Paediatrics and Child Health, University of Western Australia (UWA), Crawley, WA, Australia; 3 School of Sport Science, Exercise and Health, UWA, Crawley, WA, Australia; 4 Telethon Kids Institute, UWA, Crawley, WA, Australia

## 

Current recommendations for carbohydrate supplementation to prevent exercise-induced hypoglycaemia in individuals with type 1 diabetes (T1D) do not take into account exercise intensity or blood glucose levels during exercise. The aim of these studies was to investigate the effects of (a) exercise intensity and (b) blood glucose levels, on carbohydrate requirements to maintain stable glycaemia during exercise in individuals with T1D at basal insulin levels and to determine the glucoregulatory mechanisms underlying these effects.

Nine young adults with T1D underwent euglycaemic clamps, whereby stable blood glucose levels between 4.5 to 6mmol/L were maintained during the study at basal insulin levels. Participants performed up to 40 minutes of exercise at four different exercise intensities (35%, 50%, 65% and 80% VO_2peak_) on four separate days following a randomised counterbalanced design. In a subsequent experiment, eight participants underwent either a euglycaemic or hyperglycaemic (9.5 – 10.5mmol/L) clamp at basal insulin levels, during which they performed 40 minutes of exercise at 50% VO_2peak_, on two separate days. In both studies, glucose infusion rates (GIR) to maintain stable glycaemia were measured during exercise, constant deuterated glucose was infused to determine glucose kinetics and blood samples were collected for the analysis of glucoregulatory hormones.

The average GIR to maintain euglycaemia during exercise was 2.0±0.9, 4.0±1.5, and 4.1±1.7g/h (mean±SEM) at 35%, 50% and 65% VO_2peak_, respectively. These GIRs were all significantly greater than that at 80% VO_2peak_ where no glucose was required (p<0.05). Exercise at 80% VO_2peak_ was associated with a significant rise in catecholamine levels and endogenous glucose production (p<0.05). The average GIR to maintain stable glycaemia during exercise performed during the second experiment at 50% VO_2peak_ was similar at euglycaemia (4.9±2.1g/h) and hyperglycaemia (5.5±2.5g/h; p>0.05).

At basal insulin levels, the relationship between exercise intensity and the amount of glucose required to prevent hypoglycaemia is not linear, with no glucose required to maintain euglycaemia during high-intensity exercise. Performing moderate-intensity exercise at euglycaemia or mild hyperglycaemia does not alter the glucose requirements to maintain stable glycaemia.

